# Co-Transcriptomic Analysis of the Maize–Western Corn Rootworm Interaction

**DOI:** 10.3390/plants11182335

**Published:** 2022-09-07

**Authors:** Lise Pingault, Saumik Basu, Neetha N. Vellichirammal, William Paul Williams, Gautam Sarath, Joe Louis

**Affiliations:** 1 Department of Entomology, University of Nebraska-Lincoln, Lincoln, NE 68583, USA; 2 Corn Host Plant Resistance Research Unit, USDA-ARS, Mississippi State, MS 39762, USA; 3 Wheat, Sorghum, and Forage Research Unit, USDA-ARS, Lincoln, NE 68583, USA; 4 Department of Biochemistry, University of Nebraska-Lincoln, Lincoln, NE 68583, USA

**Keywords:** maize, Mp708, RNA-seq, Western Corn Rootworm, Transcriptome

## Abstract

The Western corn rootworm (WCR; *Diabrotica virgifera virgifera*) is an economically important belowground pest of maize. Belowground feeding by WCR is damaging because it weakens the roots system, diminishes nutrient uptake, and creates entry points for fungal and bacterial pathogens and increases lodging, all of which can significantly suppress maize yields. Previously, it was demonstrated that belowground herbivory can trigger plant defense responses in the roots and the shoots, thereby impacting intraplant communication. Although several aspects of maize-WCR interactions have been reported, co-transcriptomic remodeling in the plant and insect are yet to be explored. We used a maize genotype, Mp708, that is resistant to a large guild of herbivore pests to study the underlying plant defense signaling network between below and aboveground tissues. We also evaluated WCR compensatory transcriptome responses. Using RNA-seq, we profiled the transcriptome of roots and leaves that interacted with WCR infestation up to 5 days post infestation (dpi). Our results suggest that Mp708 shoots and roots had elevated constitutive and WCR-feeding induced expression of genes related to jasmonic acid and ethylene pathways, respectively, before and after WCR feeding for 1 and 5 days. Similarly, extended feeding by WCR for 5 days in Mp708 roots suppressed many genes involved in the benzoxazinoid pathway, which is a major group of indole-derived secondary metabolites that provides resistance to several insect pests in maize. Furthermore, extended feeding by WCR on Mp708 roots revealed several genes that were downregulated in WCR, which include genes related to proteolysis, neuropeptide signaling pathway, defense response, drug catabolic process, and hormone metabolic process. These findings indicate a dynamic transcriptomic dialog between WCR and WCR-infested maize plants.

## 1. Introduction

Western corn rootworm (WCR, *Diabrotica virgifera virgifera* LeConte) is a damaging pest of maize (*Zea mays* L.) across North America, Central and Eastern Europe [1,2]. WCR, at their larval stage, will start to damage maize roots and, more specifically, the root hairs and cortical tissues to create tunnels to reach the primary root [3,4,5,6,7]. Roots, with their root hairs, are the part of the plant that provides water and nutrient uptake and plant anchorage to soil [8]. WCR adults will feed on above ground tissues, which will impact plant photosynthesis. WCRs have developed resistance to most insecticides classes [9,10,11,12,13], crop rotation [14], and commercial maize hybrids [15,16,17,18,19] because of their ability to rapidly evolve, showing the necessity to understand maize defense mechanisms at various levels for future maize WCR resistance engineering.

Several factors contribute to plant resistance to chewing insects. For example, maize resistance to WCR can be influenced by root architecture [20], biomechanical strength [21], or biochemical composition [22]. Among direct defense, plants can adapt their morphology by increasing density of trichomes or wax composition, which can create a physical barrier [22,23,24,25]. Maize inbred lines that have been developed, such as Mp496, Mp704, and Mp708, that have a broad-based resistance to insect pests [26,27]. Both Mp704 and Mp708 have Mp496 in their background, and Mp708 was the result of the crossing of Mp704 and Tx601, which is an inbred maize line susceptible to fall armyworm (*Spodoptera frugiperda* Smith) [28].

Phytohormones also play a major role in modulating plant defenses in response to herbivore feeding [29]. For example, jasmonic acid (JA) acts as a major phytohormone in providing enhanced resistance to *Spodoptera exigua* (beet armyworm) in maize and tomato [30]. The crosstalk between JA and salicylic acid (SA) in plants in response to insect herbivory is well documented [31]. For example, chewing insects feeding on host plants activate the SA pathway, which in turn suppresses the JA-dependent defenses [32]. In the caterpillar-resistant Mp708 maize genotype, both JA and ethylene (ET) pathways were required for the accumulation of Maize Insect Resistance 1-Cysteine Protease (Mir1-CP), a key defensive protein in defense against insect pests in maize [33,34]. However, the ET pathway, uncoupled from the JA pathway, can regulate the *mir1* expression in providing enhanced defense against the phloem sap-sucking aphids [35]. Furthermore, in response to herbivory, plants activate several defense-related proteins, for example, proteases, protease inhibitors or peroxidases, which could negatively impact insect growth. Resistance to both above and belowground feeding caterpillars have been demonstrated in Mp708 plants [35,36,37,38,39,40,41]. Among the molecular mechanisms deployed by insect-resistant maize inbred lines Mp708 and Mp704, the protein Mir1-CP, as mentioned above, has been characterized as a key component for resistance to pests in maize [35,36,38,42,43]. Accumulation of Mir1-CP has been identified in maize in response to FAW and WCR infestation [38,39,44] and can damage the peritrophic matrix of caterpillars, which significantly reduces caterpillar growth [36,45]. In Mp708 maize genotype, *mir1* was expressed in root tissues at 2, 4 and 7 days after WCR infestation [38]. Belowground feeding by WCR also resulted in *mir1* transcript expression in the maize whorl tissues of Mp708, but not in susceptible Tx601 plants, indicating that WCR feeding activated defense mechanisms in Mp708 plants. Here, an RNA-seq approach was used to study the interactions between maize and WCR to investigate maize defense signaling networks from below to aboveground tissues and subsequent WCR compensatory transcriptomic responses.

## 2. Results

### 2.1. The Maize Transcriptomic Response Varies from Tissues and Time Points

**Figure 1:**
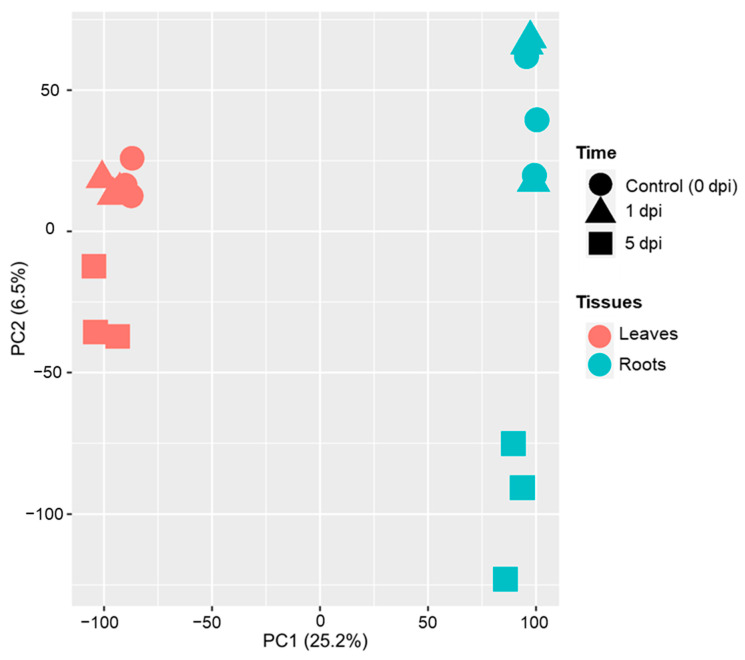
Principal component analysis (PCA) of the 35,032 genes expressed in at least one of the conditions. Conditions are represented with colors (Mp708 leaves: red, Mp708 roots: blue) and shapes (circle: 0 dpi, triangle: 1 dpi and square: 5 dpi). Each shape indicates a replicate.

To characterize the impact of WCR feeding on maize Mp708 genotype, both leaves and roots tissues were collected at 1 and 5 days post infestation (dpi) and before feeding (control; 0 dpi). Among the 39,498 annotated protein-coding genes, 35,032 (88.8%) were expressed in at least one condition. On average, 31,570 genes were expressed in each condition, ranging from 30,282 genes (Leaves: L; 0 dpi) to 32,723 (Roots: R; 0 dpi). A principal component analysis (PCA) of the 35,032 genes expressed in at least one condition was performed (Figure 1). PC1 accounting for 25.2% of the variation separated the transcriptomes by tissues (Figure 1), and PC2 accounting for 6.5% of the variance indicated a separation between 5 dpi time points and other time conditions in both leaves and roots (Figure 1). As expected, the transcriptional profile of roots was different from that of leaves.

### 2.2. Differentially Expressed Genes Vary within Tissues and Have Minimal Overlap

**Figure 2:**
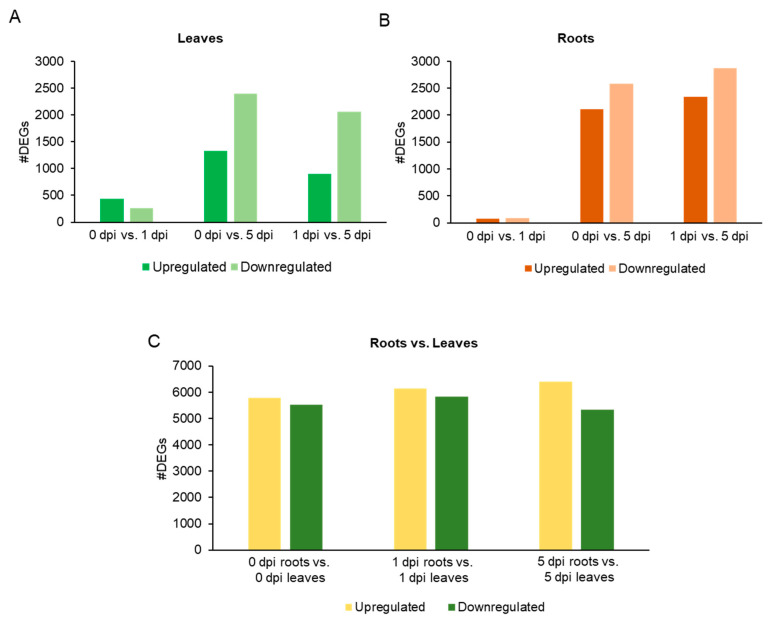
Barplot of the upregulated and downregulated differentially expressed genes (DEGs) for each tissue. (**A**) Leaves, (**B**) roots, and (**C**) roots vs. leaves for each time point.

Differentially expressed genes (DEGs) (|log_2_ (FC)| ≥ 1 and *q* value ≤ 5%) across comparisons and time points are given in Supplemental Table S1. A total of 17,383 DEGs were found for the comparisons: 0 vs. 1 dpi leaves, 0 vs. 5 dpi leaves, 1 vs. 5 dpi leaves, 0 vs. 1 dpi roots, 0 vs. 5 dpi roots, 1 vs. 5 dpi roots, 0 dpi roots vs. 0 dpi leaves, 1 dpi roots vs. 1 dpi leaves and 5 dpi roots vs. 5 dpi leaves. The least numbers of DEGs (<500) were found at 1 dpi leaves and roots (Figure 2A,B). However, more DEGs were found in leaves as compared than roots, indicating WCR herbivory of roots induced a huge transcriptional response in the leaves (Figure 2A,B). At 5 dpi, a substantial number of genes were downregulated in both leaves and roots (Figure 2A,B). A similar observation was made for the comparison of DEGs for 1 vs. 5 dpi leaves and roots (Figure 2C). These results were suggestive of significant transcriptional remodeling of the leaf (aboveground) and root (belowground) tissue transcriptomes between 1 and 5 dpi.

**Figure 3:**
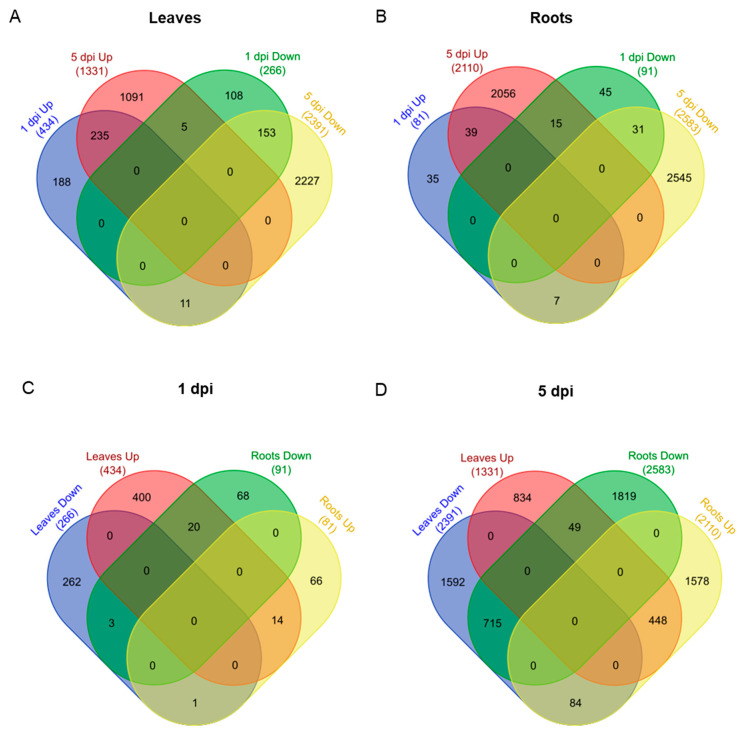
Venn diagrams of differentially expressed genes (DEGs) in Mp708 plants. The number of DEGs are indicated in parenthesis. DEGs in each tissue. (**A**) Leaves and (**B**) roots or at each time point: (**C**) 1 dpi and (**D**) 5 dpi. Up: upregulated; Down: downregulated.

The overlap of DEGs for each comparison was next analyzed for each tissue (Figure 3). A total of 434 DEGs and 1326 DEGs were upregulated in leaves at 1 and 5 dpi, respectively. Of these, 235 DEGs were shared in common between the two time points and represent 54.1% of the total number of DEGs at 1 dpi and 17.7% of the total number of DEGs at 5dpi (Figure 3A). A gene function enrichment of the 235 upregulated DEGs identified molecular functions related to: DNA-binding transcription factor activity, DNA binding or calcium ion binding (Supplemental Table S2). One hundred fifty-three genes were downregulated at 1 and 5 dpi (leaves), which represent 57.5% of the DEGs at 1 dpi (*n* = 266 genes) and 6.4% of the DEGs at 5 dpi (*n* = 2380) (Figure 3A). The gene function enrichment analysis characterized functions as: oxidoreductase activity, heme binding or peroxidase activity (Supplemental Table S2).

For the root samples, 39 and 31 genes overlapped between the root conditions 1 and 5 dpi, upregulated or downregulated, respectively (Figure 3B). The low number of genes did not allow us to perform a detailed gene function enrichment analysis. However, we examined the functions of the DEGs. Among the 39 DEGs, gene functions were related to cytochrome P450, SAUR-like auxin-responsive protein family or terpenoid cyclases/protein prenyltransferases superfamily protein (Supplemental Table S1). Among the 31 DEGs downregulated between 1 and 5 dpi, functions were related to jasmonate-zim-domain protein 11 or transcription factors (TFs) such as ERF, AP2 or G2-like (Figure 3B) (Supplemental Table S1). We also monitored the overlap of DEGs between tissues at 1 or 5 dpi (Figure 3C,D). At 1 dpi, a low number of up or downregulated genes overlapped between the two tissues. Twenty genes were upregulated in leaves and downregulated in roots (Figure 3C). At 5 dpi, a higher number of genes overlapped for upregulated (448 genes) and downregulated (715) genes between root and leaf tissues (Figure 3D).

### 2.3. Genes Were Co-Expressed in Tissues and/or Time Points

**Figure 4:**
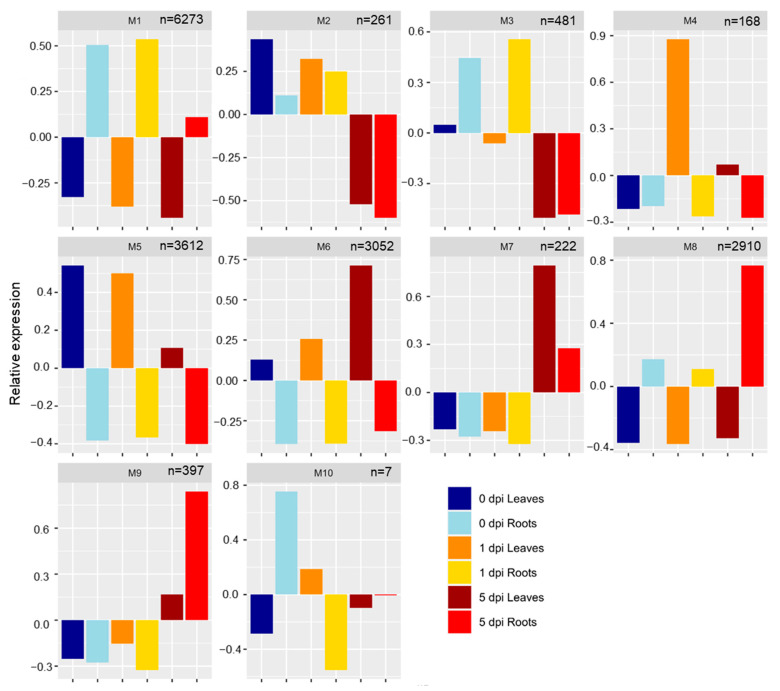
Weighted gene co-expression network analysis (WGCNA) of the 17,383 differentially expressed genes (DEGs). Barplots represent the expression pattern of the genes assigned into 10 co-expression modules (M1–10). n indicates the number of DEGs in each WGCNA module.

A weighted gene co-expression network analysis (WGCNA) of the 17,383 non-redundant DEGs was performed, and genes were sorted into 10 modules: module 1 (M1) through M10 (Figure 4) followed by gene function enrichment analysis using Kyoto Encyclopedia of Genes and Genomes (KEGG) (Supplemental Table S3). These modules could be separated by time and tissues.

#### 2.3.1. Roots: M1, M3, M8

M1 (6273 genes) and M8 (2910 genes) contained genes expressed only in roots at 0, 1, and 5 dpi (Figure 4). In M1, genes were highly expressed at 0 and 1 dpi, with lower expression at 5 dpi; however, M8 (2910 genes) had the opposite gene expression profile with higher expression of genes at 5 dpi (Figure 4). The 6273 genes included in M1 had functions related to protein localization, post-transcriptional gene silencing by RNA and positive regulation of phosphorylation. For M8, the 2910 genes were involved in regulation of the developmental process, regulation of RNA metabolite process, and cellular response to phosphate starvation processes were enriched (Supplemental Table S3). Genes that were part of M3 (481 genes) were expressed in roots at 0 and 1 dpi only (Figure 4). Gene function enrichment showed functions for M3 related to ribosome, metabolic pathways, biosynthesis of secondary metabolites or phenylpropanoid biosynthesis, all with potential roles in defense.

#### 2.3.2. Leaves: M4, M5, M6

Genes found in M4 (168 genes) were expressed only in 1 and 5 dpi leaves and were associated with regulation of cellular process, response to auxin, or regulation of nitrogen compound metabolic process (Figure 4). M5 (3612 genes) and M6 (3052 genes) were composed by genes expressed in leaves with descendant or ascendant expression profiles along the time for M5 and M6, respectively (Figure 4). Genes in M5 were enriched in functions related to biosynthesis of secondary metabolites, metabolic pathways, fatty acid biosynthesis/metabolism/elongation or porphyrin and chlorophyll metabolism, indicating downregulation of these important leaf developmental processes under WCR-induced stress. M6 DEGs were associated with the regulation of cellular process, acyl-CoA metabolic process, glycerol ether metabolic process (Supplemental Table S3). KEGG enrichment analysis revealed that genes part of M6 were enriched in functions related to circadian rhythm, photosynthesis, metabolic pathways, phenylalanine metabolism or starch and sucrose metabolism, and fatty acid biosynthesis/metabolism/elongation, porphyrin, and chlorophyll metabolism (Supplemental Table S3).

#### 2.3.3. Time Points: M2, M7, M9, M10

M2 comprised 261 genes expressed at 0 and 1 dpi in both tissues (Figure 4). Biological process analysis showed gene enrichment related to translation synthesis, β-glucan metabolism/biosynthesis process, spindle checkpoint, NADPH regeneration for M2 (Supplemental Table S3). Genes that are part of M7 (222 genes) and M9 (397 genes) were upregulated in both leaves and roots at 5 dpi (Figure 4). Genes comprising these modules have functions related to cell communication and defense mechanisms. M7 was composed of genes with functions related to the regulation of catalytic activity, regulation of protein metabolic process, regulation of cell communication, regulation of phosphorus metabolic process, and regulation of response to stimulus, which indicate that both tissues respond to WCR attack by expressing genes involved in defense mechanisms. Genes in M9 had functions related to the regulation of RNA metabolic process, regulation of nitrogen compound, biosynthetic process, phosphorus metabolic process, programmed cell death, sucrose metabolic process. M10 had genes upregulated in uninfested roots and leaves at 1 dpi, with functions related to nucleoside phosphate biosynthetic process, monovalent inorganic cation transport or ATP biosynthetic process.

### 2.4. Modulations in Maize Hormone Pathways during WCR Infestation

**Figure 5:**
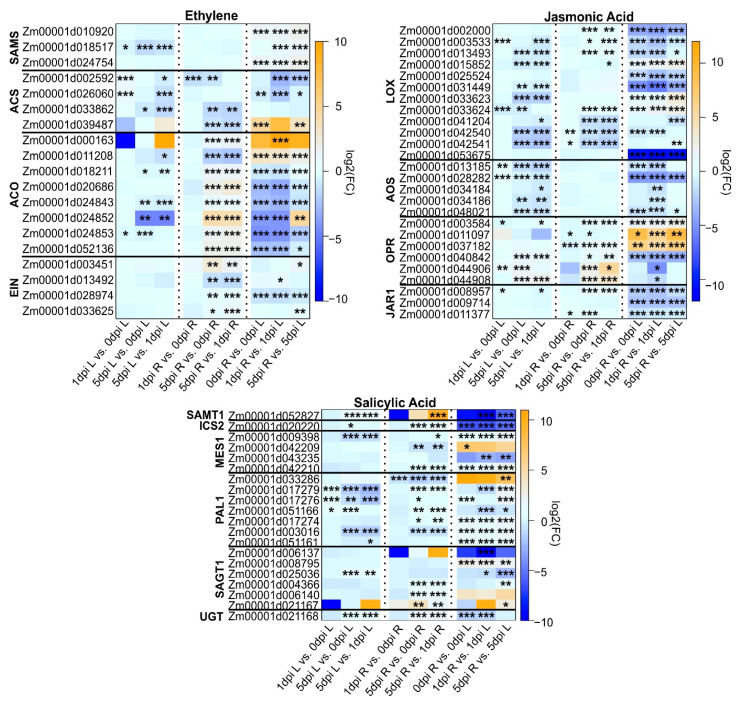
Heatmap of genes encoding plant hormonal pathways related to ethylene (ET), jasmonic acid (JA), and salicylic acid (SA). Each column corresponds to a condition for each tissue (L: leaves or R: roots). Each cell contains the corresponding log_2_ (fold-change (FC Infested/Uninfested); genes upregulated are indicated in orange and downregulated in blue) and adjusted *p* value (*** < 0.001, 0.001 < ** < 0.01, 0.01 < * < 0.05).

The transcriptomic activity of genes associated with hormones involved in plant defenses [46] was investigated (Figure 5).

#### 2.4.1. Ethylene (ET)

There are five majors steps for the synthesis of ET [47]. Nineteen genes involved in ET pathway were differentially expressed between at least two conditions and could be divided into four gene families. SAMS (S-adenosyl-L-methionine synthase) genes were downregulated within the leaf contrasts but upregulated in 1 and 5 dpi roots compared to leaves (Figure 5). *ACS* (ACC synthase) genes were downregulated for all the comparisons, except for one gene *Zm00001d039487*, which was upregulated in two contrasts. Among the *ACO* (ACC oxidase) genes, six of the eight DEGs were downregulated in uninfested roots compared to uninfested leaves, and within leaf comparisons (Figure 5). Significant changes in *ACO* gene abundances were seen in the 1 and 5 dpi root comparisons. These differences were not observed in 1 dpi versus 0 dpi root comparisons. When compared to leaves, six and two *ACO* genes were down and upregulated, respectively, in the 1 dpi comparisons, and five and two down- and upregulated, respectively, in the 5 dpi comparisons (Figure 5). In *Arabidopsis thaliana*, *EIN2* is required for ethylene signaling [48]. Here, four genes of the EIN family were DEGs and generally downregulated across all contrasts, except for *Zm00001d003451*, which was significantly enriched in roots at 5 dpi compared to its control (Figure 5).

#### 2.4.2. Jasmonic Acid (JA)

α-linolenic acid (18:3) is the starting point for JA biosynthesis and acts as a substrate for lipoxygenases (LOX). The *LOX* gene family in maize contains 13 loci (*ZmLOX1–13*) [49]. Further, LOX enzymes were subdivided into two groups, namely 9-lipoxygenases and 13-lipoxygenases, depending on where they oxygenate α-linolenic acid. There are seven 9-lipoxygenases (*ZmLOX1*, 2, 3, 4, 5, 6, 12) and six 13-lipoxygenases (*ZmLOX7*, 8, 9, 10, 11, 13) in maize. 13-lipoxygenases catalyze the first step to the production of JA, while products of 9-lipoxygenases can still have defensive functions against insect herbivory [50]. Products of LOX catalysis are progressively converted by allene oxide synthase (AOS), allene oxide cyclase (AOC), and oxophytodienoic acid reductase (OPR), ultimately resulting in JA production [51,52]. Genes encoding AOS, LOX, and JAR1 (which conjugate JA to isoleucine [53]) were downregulated between all comparisons, except for transcripts for three *LOX* genes that were significantly enriched in the 5 dpi root/leaf comparisons (Figure 5). Genes encoding *OPR* were downregulated in leaves across all comparisons, and transcripts of two *OPR* genes that were enriched in the control root/leaf comparisons remained enriched in the 1 and 5 dpi root versus leaves contrast. Notably, two other *OPR* genes were upregulated at 5 days in roots relative to the expression in 1-day roots. Maize *OPR7* and *OPR8* function in the conversion of OPDA to JA [54]. Additionally, it was shown that the Mp708 plants had elevated constitutive levels of JA [40,55]. Our results also demonstrate that the Mp708 roots had constitutive expression of *OPR7* and *OPR8*, and these levels remained elevated and unchanged throughout the 5-day infestation of WCR (Supplemental Table S1). These results align with the previous findings that the Mp708 plants have constitutive accumulation of JA.

#### 2.4.3. Salicylic Acid (SA)

We evaluated the genes involved in SA biosynthesis, as SA is often implicated in defense against pests. Biosynthesis of SA can take place via the isochorismate (IC) and/or phenylalanine ammonia-lyase (PAL) pathways [56,57]. In the IC pathway, IC produced in chloroplasts from chorismate by ICS is eventually converted to SA in the cytosol, whereas chorismate is exported from the chloroplasts to the cytosol and is used for the biosynthesis of phenylalanine. Phenylalanine is the primary precursor for the phenylpropanoid pathway and is converted to 4-cinnamic acid by PAL (phenylalanine ammonia lyase). 4-cinnamic acid is subsequently converted to benzoic acid, and ultimately to SA [54]. SA can be methylated by SAMTs to form methyl salicylate or conjugated to sugars by glycosyl transferases to form SA-sugar derivatives that are stored in vacuoles. Glycosidases can convert the SA-glycosyl esters back to free SA. Methylesterases (MES1) can convert SA methyl ester back to free SA and play a role of increasing cellular SA levels. *SAMT1* was generally downregulated except in the 5 versus 1 dpi root comparisons. One copy of *ICS2* was part of the DEGs and was downregulated in all comparisons (Figure 5). Four copies of putative methylesterases, *MES1*, were found, of which three were downregulated in all comparisons, and one *Zm00001d042209* appeared to be more enriched in roots as compared to leaves. One *PAL* gene appeared to be more enriched in roots relative to leaves, whereas all the other copies were downregulated across comparisons (Figure 5). Similarly, many of the potential SA glycosyltransferases (*SAGT1*) were downregulated across comparisons, except for two that showed a mild enrichment in the roots. A UGT gene that was part of the DEGs was also downregulated in all comparisons (Figure 5).

### 2.5. Maize DIMBOA Pathway Was Turned Off after Extended WCR Feeding

**Figure 6:**
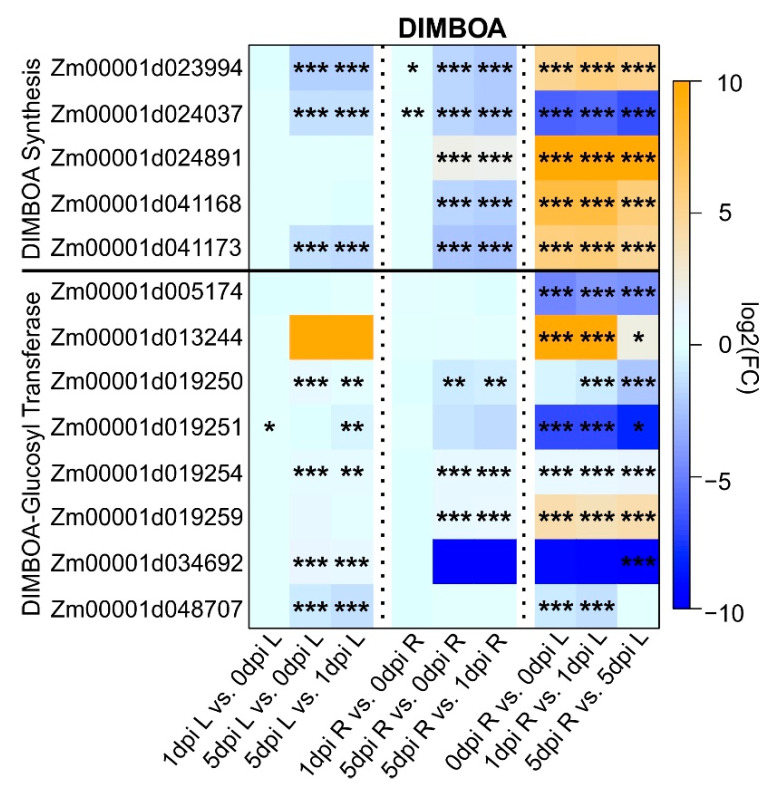
Heatmap of differentially expressed genes (DEGs) associated with DIMBOA biosynthesis pathway in maize. Each column corresponds to a condition for each tissue (L: leaves or R: roots). Each cell contains the corresponding log_2_ (fold-change (FC Infested/Uninfested); genes upregulated are indicated in orange and downregulated in blue) and adjusted *p* value (*** < 0.001, 0.001 < ** < 0.01, 0.01 < * < 0.05). Maize gene IDs for the DIMBOA pathway were obtained from the maizeGDB database [62].

Benzoxanoids are secondary metabolites that act as natural pesticides. Their biosynthesis involves nine enzymes to form a linear pathway leading to the storage of DIMBOA as glucoside conjugate. Here, we identified DEGs encoding proteins involved in the synthesis of DIMBOA and DIMBOA-glucoside [58,59,60] (Figure 6). Out of the five genes associated with DIMBOA biosynthesis, transcripts for four were more enriched on roots relative to leaves across all comparisons, and one was downregulated, suggesting greater DIMBOA biosynthetic capacity in roots (Figure 6). DIMBOA is glycosylated by several glucosyl transferases (GTs) using UDPG as a substrate to produce DIMBOA-ß-D-glucoside and UDP. Among the 14 GTs that can catalyze this reaction [61], eight were differentially expressed. In roots, two genes (*Zm0001d019254* and *Zm00001d019259*) were upregulated and one gene (*Zm00001d019250*) was downregulated for the comparison 0 vs. 5 dpi and 1 vs. 5 dpi (Figure 6). For the roots, three genes (*Zm00001d029250*, *Zm00001d019254* and *Zm00001d034692*) were upregulated at 0 vs. 5 dpi and 1 vs. 5 dpi. Only one gene was upregulated at 0 vs. 1 dpi (*Zm00001d019251*) and downregulated at 1 vs. 5 dpi (Figure 6).

### 2.6. WCR Transcriptome Is Remodeled between 1 and 5 dpi

**Figure 7:**
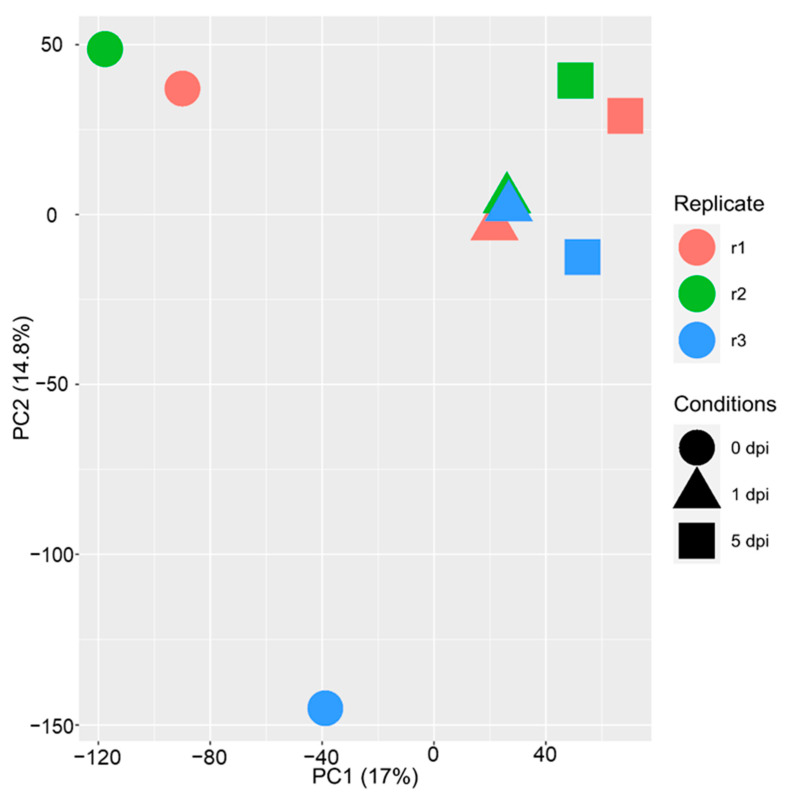
Principal component analysis (PCA) of the 19,358 genes expressed in WCR. Conditions are represented with colors (replicate 1: red, replicate 2: green and replicate 3: blue) and shapes (circle: 0 dpi, triangle: 1 dpi and square: 5 dpi).

**Figure 8:**
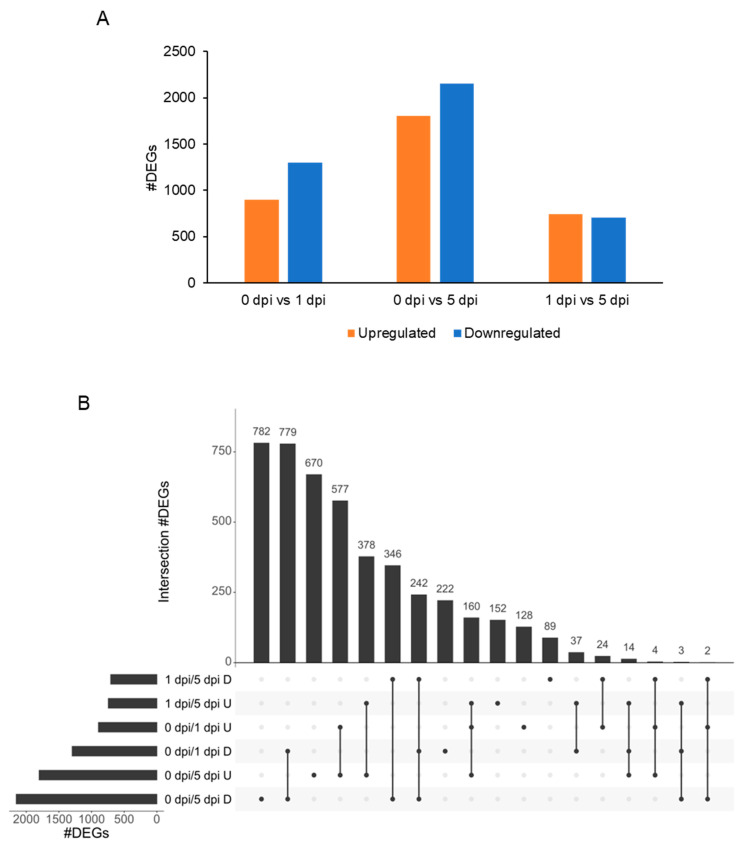
Organization of the 4609 WCR differentially expressed genes (DEGs). (**A**) Barplot of the number of upregulated (orange) and downregulated (blue) DEGs. (**B**) UpSet intersection plot of DEGs. Intersections are represented by a line connected to one or more dots. The number of common DEGs are indicated at the top of each bar, and conditions are organized according to the number of DEGs. D, downregulated in cond2; U, upregulated in cond2 when cond1/cond2.

Complementary to the maize transcriptome, we also investigated the WCR transcriptome before infestation (0 h), and after infestation at 1 and 5 dpi. A PCA analysis of the 19,358 genes expressed in at least one condition showed that samples were separated according to the time course (PC1, 17%), indicating differential regulation of transcription following feeding (Figure 7). In total, 4609 genes were differentially expressed (Supplemental Table S4). At 1 and 5 dpi, a higher number of genes were downregulated (Figure 8). Furthermore, the number of up- or downregulated genes between conditions 1 and 5 dpi were comparable (Figure 8A). We investigated the function of the up- and downregulated WCR genes for each comparison (Supplemental Table S4). At 1 dpi, the 1297 downregulated genes had functions related to defense response, response to biotic stimulus, response to other organisms, and response to external stimulus. In contrast, the 895 upregulated genes had functions related to the monocarboxylic acid metabolic process, cellular metabolic compound salvage, fatty acid metabolic process, cellular compound organization, or cytoskeleton organization (Supplemental Table S5). At 5 dpi, 2154 downregulated genes had functions linked to proton transmembrane transport, translation, peptide biosynthetic process, organonitrogen compound biosynthetic process, cation transport or defense response. We also functionally characterized the DEGs between conditions 1 and 5 dpi. The 707 downregulated genes at 5 dpi had functions related to defense response, response to stimulus signal transduction, cell communication, while genes upregulated at 5 dpi were associated with functions as DNA replication, DNA metabolic process, post-transcriptional gene silencing by RNA.

#### 2.6.1. 0 h vs. 1 dpi

Eighteen genes were upregulated at 1 dpi compared to 0 h (FPKM_0dpi_ = 0). These genes were related to functions classified as bromodomain-containing protein, phosphatidylinositol 4-kinase alpha-like, Krueppel-like factor 12, epidermal growth factor receptor substrate 15-like 1, asparagine synthetase domain-containing (Supplemental Table S5). Genes upregulated at 1 dpi compared to 0 h and expressed in control condition (877 genes) were related to functions: calphotin-like, endoglucanase-like, chaoptin-like, venom peptide HsVx1-like or cuticle protein LPCP-23-like (Supplemental Table S5). Downregulated genes were associated with functions related to acaloleptin A-like, glycine-rich protein-like, cecropin-B2-like or acidic mammalian chitinase-like.

#### 2.6.2. 0 h vs. 5 dpi

Twelve DEGs were found upregulated at 5 dpi, but not expressed at 1 dpi or 0 h (FPKM_0dpi_ = 0 and FPKM_1dpi_ = 0). The functions of these genes were linked to: ctenidin-1-like, cuticle protein LPCP-23-like, neuropeptide-like 3, glucose dehydrogenase, neuropeptide-like 3, putative gustatory receptor 28 b (Supplemental Table S5). Genes expressed at 5 dpi but not expressed in control (1759 DEGs) have functions related to: bromodomain-containing protein, neuropeptide-like 3, prisilkin-39-like, phosphatidylinositol 4-kinase alpha-like, elongation of long-chain fatty acids protein, cuticle protein or proline-rich protein (Supplemental Table S5). Functions of downregulated genes (2154 DEGs) were associated with: alpha-crystallin B chain-like, thaumatin-like protein 1 b, tenecin-1-like, venom serine protease inhibitor-like, cathepsin L-like proteinase, putative glucosylceramidase 3, acaloleptin A-like, drosomycin-like or venom allergen 3-like (Supplemental Table S5). In addition, due to the overlap of the DEGs, a large number of genes up- or downregulated were shared between the comparisons: 0 vs. 1 dpi, 0 vs. 5 dpi or 1 vs. 5 dpi (Figure 8B). At 5 dpi, 782 genes were downregulated, whereas 777 genes were downregulated at 5 and 1 dpi when compared with the controls (0 dpi). Further, the gene function enrichment analysis identified functions related to cellular component organization, actin cytoskeleton organization, protein transport, or lipid biosynthetic process (Figure 8B, Supplemental Table S5). Overlapping genes downregulated or upregulated for the three comparisons indicate that these genes are differentially expressed during the experimental time course. Here, 242 genes were downregulated and 160 genes were upregulated within the three comparisons 0 vs. 1 dpi, 0 vs. 5 dpi and 1 vs. 5 dpi (Figure 8B). Gene function enrichment analysis showed that the 242 downregulated genes were associated with defense response, innate immune response, reproductive behavior, mating behavior, or visual perception (Supplemental Table S5). The 160 upregulated genes have functions related to DNA replication or DNA metabolic process. Three hundred and forty six genes were downregulated between 1 vs. 5 dpi and 0 vs. 5 dpi, suggesting that these genes were not impacted at 1 dpi (Figure 8B). These genes were involved in proteolysis, neuropeptide signaling pathway, defense response, drug catabolic process, and hormone metabolic process.

## 3. Discussion

Plant metabolism is impacted in response to biotic stress, and intraplant communication plays a crucial role in this process. We have previously shown that maize plants subjected to leaf herbivory by the European corn borer (ECB; *Ostrinia nubilalis*) were more resistant to WCR larval feeding on the roots [41]. In addition, the analysis of the maize root transcriptomes of plants infested with ECB indicated an upregulation of genes with functions related to phytohormone or defense in root tissues. Here, we show that the WCR attack remodels the transcriptomes of both below and aboveground maize tissues. In addition, we highlighted the changes of the WCR transcriptome during the time-course of this study.

A previous study reported no overlap of DEGs between roots and leaves tissues after pest infestation [63]; here, we observed an overlap between up- and downregulated genes between leaves and roots at 5 dpi in WCR-infested plants relative to 0 dpi. Genotype differences and age of WCRs used in our study and that of Erb et al. [63] could account for this disparity. Nevertheless, our results indicated a strong transcriptomic response with common gene activation in below and aboveground tissues with increasing time of WCR feeding on maize Mp708 roots. For both leaves and roots tissues, we found a higher transcriptional response at 5 dpi compared to other time points indicating that response to WCR feeding built up over time.

Maize Mp708 line was defined as a resistant line in response to insect feeding associated with the expression of *mir1*. Here, *mir1* (*Zm00001d036542*) was expressed in both roots and leaves tissues, but the expression of *mir1* was constitutive in the leaves at all the time points (0, 1, and 5 dpi), whereas *mir1* was upregulated in the root at 5 dpi compared to other time points (Supplemental Figure S1). This result suggested that maize defense mechanisms were activated after the initiation of WCR feeding and is not an immediate effect. The accumulation of *mir1* transcripts was previously linked with JA and ET levels in Mp708 in response to fall armyworms [34]. Here, we showed that genes involved in the ET pathway are upregulated in root tissues, and their expression increased over time, indicating that ET plays an important role in defense response in Mp708 against WCR herbivory. Both ET and JA are well documented for their defensive roles during plant responses to insect herbivory [34,35,47,64,65]. Here, we have shown that genes related to SA pathway were mainly downregulated. SA and JA were previously described with an antagonistic action, but not all the time. In Arabidopsis, it was also demonstrated that both SA and JA act synergistically to promote effector-triggered immunity against *Pseudomonas syringae* pv. *maculicola* [66]. Because Mp708 plants had elevated levels of JA [40,55], higher constitutive expression of *OPR7*, a gene involved in the conversion of OPDA to JA, remained high before and after WCR feeding for 7 days [38]. Similarly, our results also demonstrated elevated constitutive expression of *OPR7* and *OPR8* genes in Mp708 roots before and after WCR feeding for 5 days (Supplemental Table S1). However, a recent study by Ye et al. (2022) [67], showed that the JA biosynthesis genes were upregulated in maize roots after WCR feeding for 72 h. The constitutive and induced expression of JA biosynthesis genes in the maize genotypes used in our study and in Ye et al. [67] may contribute to the discrepancy between results in maize-WCR interactions. In the future, a metabolomic approach would benefit to validate our transcriptomic results.

In addition to phytohormones, plants also use secondary metabolites as a defense mechanism. Among these metabolites, maize plants store benzoxazinoids in a non-toxic form when they are not injured, but tissue injury results in benzoxazinoids breaking down into toxic compounds [68]. However, these benzoxazinoids can be redirected and controlled by the herbivores and can be used as self-defense for WCR against their natural predators [69]. In roots, we found that the DIMBOA-synthesis genes were downregulated, while genes associated with DIMBOA-glucosyl transferase and DIMBOA-glucoside dehydrogenase were mostly upregulated. Collectively, our results suggest that the expression of genes related to benzoxazinoids in WCR resistant maize plant were altered after WCR herbivory.

The transcriptomic analysis of Mp708 revealed that after 1 day post infestation, expression profiles of genes with functions related to cell wall organization, cellulose metabolic process, or biosynthetic process were unchanged. However, their expression was drastically decreased after 5 dpi, suggesting that it takes at least 5 days post-WCR infestation to start affecting plant tissues and physiology. It is plausible that the newly hatched WCR larvae did not actively feed on the maize roots at the beginning (1 dpi), but as the days progressed (5 dpi), the late instar WCR larvae were able to modulate physiological changes in the plant tissues. Alternatively, it is equally likely that the “cues” released from late instar WCR larvae were able to suppress the maize physiological responses. Similarly, the transcription profile of *mir1* revealed no change in the leaf, but the expression in the roots increased significantly at 5 dpi. These data suggest that there is a fine tuning of intraplant defense, providing resources to regions under active herbivory (roots) and not to distal sites (leaves).

Similar to the WCR herbivory affecting plant transcriptomes, WCR transcriptomes were also impacted after feeding for 1 and 5 days on maize Mp708 plants. Changes in the WCR transcriptome were broadly related to their development and counteracting plant defense mechanisms. At 1 dpi, upregulated genes compared to before infestation had functions related to larval growth and contained many uncharacterized genes. A large number of WCR DEGs with unknown functions highlight the need for further annotation of the WCR genome. As mentioned before, plant defense mechanisms were activated in maize roots at 5 dpi. This activation was also reflected in the WCR transcriptome with the suppression of the gene transcription associated with functions related to allergen or defense. Some of these WCR genes that were previously reported to be downregulated in the WCR larval transcriptome in response to two maize toxins Cry34/35Ab1 [70,71] were also downregulated in our study. These genes included zinc ion binding (downregulated at 1 and 5 dpi), lipase activity (downregulated at 1 dpi and upregulated at 5 dpi), lipase activity (upregulated at 5 dpi and 1 dpi) or upregulated cell communication (upregulated 0 dpi vs. 5 dpi) [72,73,74]. These results demonstrate that the plant defenses in a resistant maize genotype can negatively impact all the developmental stages of WCR.

## 4. Materials and Methods

### 4.1. Insect Growth

WCR eggs were obtained from Crop Characteristics, Inc., Farmington, MN, and were maintained as described previously [41]. Briefly, the WCRs used in this study were originally collected from susceptible non-Bt maize fields in Minnesota, United States. This colony was further maintained on non-Bt maize plants at Crop Characteristics Inc. for several generations (>80). The WCR eggs obtained were kept in growth chamber with 14:10 (L:D) h photoperiod at 23 °C for hatching. Newly hatched neonate larvae were used for the experiments.

### 4.2. Plant Growth and WCR Infestations

Mp708 plants were grown in 3.8 cm × 21.0 cm plastic Cone-tainers (Hummert International, Earth City, MO, USA) containing soil mixed with vermiculite and perlite (PRO-MIX BX BIOFUNGICIDE + MYCORRHIZAE, Premier Tech Horticulture Ltd., Olds, AB, Canada) in growth chambers with 14:10 (L:D) h photoperiod, 160 μE m^−2^ s^−1^, 25 °C, and 50–60% relative humidity. All plants for the experiments were used at the V2–V3 developmental stage (~2 weeks) [75]. Maize roots were infested with ten newly hatched neonate WCR larvae [41] and plant tissues (both roots and leaves) were collected at 1 and 5 day post infestation (dpi). For leaf sample collection, the tissues in the yellow-green region of the whorl (where the caterpillars normally feed) were harvested. For the root tissue collection, ~10 cm from the root–shoot junction, which forms the middle region of the entire root system, was harvested, as described previously [76]. WCR uninfested plants were used as controls. In addition, WCR used to infest the maize plants were collected at 1 and 5 dpi and before infestation (i.e., 0 dpi, which are the newly hatched larvae that were never exposed to Mp708 roots). In total, three replicates were collected for each experimental condition. For each replicate, three infested or uninfested leaves and root tissues and WCR were collected and flash-frozen in liquid nitrogen.

### 4.3. RNA Extraction and RNA-Seq Libraries Construction and Sequencing

Maize and WCR tissues (80–100 mg) were ground using 2010 Geno/Grinder^®^ (SPEX SamplePrep, Metuchen, NJ, USA) for 40 s at 1400 strokes min^−1^. Total RNA was extracted from the homogenized tissue using Qiagen RNeasy Plant Mini Kit. Extracted total RNA was quantified through Nanodrop 2000c Spectrophotometer (Thermo Fisher Scientific, Waltham, MA, USA). Then, RNA-seq libraries were constructed by using the mRNA-seq standard TruSeq protocol from Illumina. RNA-seq libraries were sequenced to produce 50 bp paired-end reads. Each sample had an average of 20 million reads. The transcriptomics dataset is available under Bioproject: PRJNA781637.

### 4.4. Analysis of RNA-Seq Libraries

The quality check of the RNA-seq libraries was performed with FastQC [77], and reads with a Phred score lower than 20 and length below 45 base pairs were removed with Trimmomatic v0.39 [78]. For the maize tissues, trimmed reads were mapped to the maize reference genome v4 (genotype B73: https://phytozome-next.jgi.doe.gov/info/Zmays_RefGen_V4, accessed on 30 May 2022) with Tophat2 [79] using the following parameters: 1 mismatch (-N 1), 0 splicing mismatch (-m 0), unique mapped reads (-g 1 -M). For the WCR, reads after trimming were mapped to the reference genome Dvir_v2.0 (https://www.ncbi.nlm.nih.gov/assembly/GCF_003013835.1/, accessed on 30 May 2022) with Tophat2 [79] used for the mapping with the following parameters: 0 mismatch (-N 0), 0 splicing mismatch (-m 0), unique mapped reads (-g 1 -M). The transcript reconstruction was performed with Cufflinks v2.2.1 with the following parameters: quantification against the reference annotation only (-G), multi-read-correct (-u) and frag-bias-correct (-b). The differential expressed gene analysis was performed with Cuffdiff 2.2.1. Differentially expressed genes (DEGs) were identified with the following parameters: *q* values ≤ 5% and |log_2_ (Infested/Control)| ≥ log_2_ (2). Co-expression modules were identified by the weighted gene co-expression network (WGCNA) [80]. Genes involved in JA, ET and SA pathways for maize have been described in our previous publication [81]. Maize gene IDs for the DIMBOA pathway were obtained from the maizeGDB database [62].

### 4.5. Functional Annotation

The GOBU package was used for enrichment calculations [82]. The full set of maize gene annotation was used as the reference comparison set against down- or upregulated DEGs. The *p* values were calculated using Fisher’s exact test and were corrected for multiple testing with FDR method using the R module called ‘*p*-adjust’.

## 5. Conclusions

The transcriptomic analysis of maize above and belowground tissues after WCR infestation indicated that there are changes in the gene expression both at the site of WCR feeding and tissues distal to WCR feeding (i.e., leaves). Plant transcriptomic responses modulated over time and were higher at 5 than at 1 dpi. Based on our data and previous findings, it is highly likely that the resistance mechanism in the Mp708 genotype is a multi-trait phenotype, and Mir1-CP could act as a central defensive protein involved in imparting pest resistance. Determining the role of Mir1-CP-dependent protein networks or other defense proteins connected to Mir1-CP that contribute to above- and belowground defense signaling is critical to understand global and tissue-specific defenses in the insect-resistant maize genotype. Additionally, we also observed changes to the WCR transcriptome with the deactivation of defense genes after 5 dpi. Understanding the major maize defense signaling networks and compensatory responses in WCR to combat plant defenses could lead to the development of novel pest management strategies.

## Data Availability

The transcriptomics dataset is available under Bioproject: PRJNA781637.

## Supplementary Materials

The following supporting information can be downloaded at: https://www.mdpi.com/article/10.3390/plants11182335/s1, Supplemental Table S1: Contrast summary for the genes expressed in maize roots and root tissues at 1 dpi and 5 dpi. Supplemental Table S2: Gene function enrichment output from Venn Diagram. Supplemental Table S3: Gene function enrichment output for each module. Supplemental Table S4: Contrast summary for the genes expressed in WCR tissues at 1 dpi and 5 dpi. Supplemental Table S5: Gene function enrichment for WCR. Supplemental Figure S1: The expression level of *mir1* (*Zm00001d036542*) across each maize conditions.

## References

[1] Dun Z., Mitchell P.D., Agosti M. (2010). Estimating *Diabrotica virgifera virgifera* Damage Functions with Field Trial Data: Applying an Unbalanced Nested Error Component Model. J. Appl. Entomol..

[2] Tinsley N.A., Estes R.E., Gray M.E. (2013). Validation of a Nested Error Component Model to Estimate Damage Caused by Corn Rootworm Larvae. J. Appl. Entomol..

[3] Urías-López M.A., Meinke L.J. (2001). Influence of Western Corn Rootworm (Coleoptera: Chrysomelidae) Larval Injury on Yield of Different Types of Maize. J. Econ. Entomol..

[4] Branson T. (1986). Larval Feeding Behavior and Host—Plant Resistance in Maize. Methods for the Study of Pest Diabrotica.

[5] Chiang H.C. (1973). Bionomics of the Northern and Western Corn Rootworms. Annu. Rev. Entomol..

[6] Riedell W.E. (1990). Rootworm and Mechanical Damage Effects on Root Morphology and Water Relations in Maize. Crop Sci..

[7] Riedell W., Kim A. (1990). Anatomical Characterization of Western Corn Rootworm Damage in Adventitious Roots of Maize. J. Iowa Acad. Sci..

[8] Eshel A., Beeckman T. (2013). Plant Roots: The Hidden Half.

[9] Ball H.J., Weekman G.T. (1962). Insecticide Resistance in the Adult Western Corn Rootworm in Nebraska. J. Econ. Entomol..

[10] Meinke L.J., Siegfried B.D., Wright R.J., Chandler L.D. (1998). Adult Susceptibility of Nebraska Western Corn Rootworm (Coleoptera: Chrysomelidae) Populations to Selected Insecticides. J. Econ. Entomol..

[11] Parimi S., Meinke L.J., Wade French B., Chandler L.D., Siegfried B.D. (2006). Stability and Persistence of Aldrin and Methyl-Parathion Resistance in Western Corn Rootworm Populations (Coleoptera: Chrysomelidae). Crop Prot..

[12] Pereira A.E., Wang H., Zukoff S.N., Meinke L.J., French B.W., Siegfried B.D. (2015). Evidence of Field-Evolved Resistance to Bifenthrin in Western Corn Rootworm (*Diabrotica virgifera virgifera* LeConte) Populations in Western Nebraska and Kansas. PLoS ONE.

[13] Zhu K.Y., Wilde G.E., Higgins R.A., Sloderbeck P.E., Buschman L.L., Shufran R.A., Whitworth R.J., Starkey S.R., He F. (2001). Evidence of Evolving Carbaryl Resistance in Western Corn Rootworm (Coleoptera: Chrysomelidae) in Areawide-Managed Cornfields in North Central Kansas. J. Econ. Entomol..

[14] Levine E., Spencer J.L., Isard S.A., Onstad D.W., Gray M.E. (2002). Adaptation of the Western Corn Rootworm to Crop Rotation: Evolution of a New Strain in Response to a Management Practice. Am. Entomol..

[15] Gassmann A.J., Petzold-Maxwell J.L., Keweshan R.S., Dunbar M.W. (2011). Field-Evolved Resistance to Bt Maize by Western Corn Rootworm. PLoS ONE.

[16] Gassmann A.J., Shrestha R.B., Jakka S.R.K., Dunbar M.W., Clifton E.H., Paolino A.R., Ingber D.A., French B.W., Masloski K.E., Dounda J.W. (2016). Evidence of Resistance to Cry34/35Ab1 Corn by Western Corn Rootworm (Coleoptera: Chrysomelidae): Root Injury in the Field and Larval Survival in Plant-Based Bioassays. J. Econ. Entomol..

[17] Gassmann A.J., Shrestha R.B., Kropf A.L., Clair C.R.S., Brenizer B.D. (2020). Field-Evolved Resistance by Western Corn Rootworm to Cry34/35Ab1 and Other *Bacillus thuringiensis* Traits in Transgenic Maize. Pest Manag. Sci..

[18] Ludwick D.C., Meihls L.N., Ostlie K.R., Potter B.D., French L., Hibbard B.E. (2017). Minnesota Field Population of Western Corn Rootworm (Coleoptera: Chrysomelidae) Shows Incomplete Resistance to Cry34Ab1/Cry35Ab1 and Cry3Bb1. J. Appl. Entomol..

[19] Zukoff S.N., Ostlie K.R., Potter B., Meihls L.N., Zukoff A.L., French L., Ellersieck M.R., Wade French B., Hibbard B.E. (2016). Multiple Assays Indicate Varying Levels of Cross Resistance in Cry3Bb1-Selected Field Populations of the Western Corn Rootworm to MCry3A, ECry3.1Ab, and Cry34/35Ab1. J. Econ. Entomol..

[20] Branson T.F., Sutter G.R., Fisher J.R. (1982). Comparison of a Tolerant and a Susceptible Maize Inbred Under Artificial Infestations of *Diabrotica virgifera virgifera*: Yield and Adult Emergence. Environ. Entomol..

[21] Meihls L.N., Kaur H., Jander G. (2012). Natural Variation in Maize Defense against Insect Herbivores. Cold Spring Harb. Symp. Quant. Biol..

[22] Van Dam N.M. (2009). Belowground Herbivory and Plant Defenses. Annu. Rev. Ecol. Evol. Syst..

[23] War A.R., Taggar G.K., Hussain B., Taggar M.S., Nair R.M., Sharma H.C. (2018). Plant Defence against Herbivory and Insect Adaptations. Aob Plants.

[24] Levin D.A. (1973). The Role of Trichomes in Plant Defense. Q. Rev. Biol..

[25] Tian D., Tooker J., Peiffer M., Chung S.H., Felton G.W. (2012). Role of Trichomes in Defense against Herbivores: Comparison of Herbivore Response to Woolly and Hairless Trichome Mutants in Tomato (*Solanum lycopersicum*). Planta.

[26] Williams W.P., Davis F.M. (1982). Registration of Mp704 Germplasm Line of Maize (Reg. No. GP116). Crop Sci..

[27] Williams W.P., Davis F.M., Windham G.L. (1990). Registration of Mp708 Germplasm Line of Maize. Crop Sci..

[28] Williams P.W., Buckley P.M., Davis F.M. (1985). Larval Growth and Behavior of the Fall Armyworm (Lepidoptera: Noctuidae) on Callus Initiated from Susceptible and Resistant Corn Hybrids. J. Econ. Entomol..

[29] Erb M., Meldau S., Howe G.A. (2012). Role of Phytohormones in Insect-Specific Plant Reactions. Trends Plant Sci..

[30] Al-Zahrani W., Bafeel S.O., El-Zohri M. (2020). Jasmonates Mediate Plant Defense Responses to *Spodoptera exigua* Herbivory in Tomato and Maize Foliage. Plant Signal Behav..

[31] Thaler J.S., Humphrey P.T., Whiteman N.K. (2012). Evolution of Jasmonate and Salicylate Signal Crosstalk. Trends Plant Sci..

[32] Chung S.H., Rosa C., Scully E.D., Peiffer M., Tooker J.F., Hoover K., Luthe D.S., Felton G.W. (2013). Herbivore Exploits Orally Secreted Bacteria to Suppress Plant Defenses. Proc. Natl. Acad. Sci. USA.

[33] Harfouche A.L., Shivaji R., Stocker R., Williams P.W., Luthe D.S. (2006). Ethylene Signaling Mediates a Maize Defense Response to Insect Herbivory. MPMI.

[34] Ankala A., Luthe D.S., Williams W.P., Wilkinson J.R. (2009). Integration of Ethylene and Jasmonic Acid Signaling Pathways in the Expression of Maize Defense Protein Mir1-CP. Mol. Plant Microbe Interact..

[35] Louis J., Basu S., Varsani S., Castano-Duque L., Jiang V., Williams W.P., Felton G.W., Luthe D.S. (2015). Ethylene Contributes to *Maize Insect Resistance1*-Mediated Maize Defense against the Phloem Sap-Sucking Corn Leaf Aphid. Plant Physiol..

[36] Pechan T., Ye L., Chang Y., Mitra A., Lin L., Davis F.M., Williams W.P., Luthe D.S. (2000). A Unique 33-KD Cysteine Proteinase Accumulates in Response to Larval Feeding in Maize Genotypes Resistant to Fall Armyworm and Other Lepidoptera. Plant Cell.

[37] Gill T.A., Sandoya G., Williams P., Luthe D.S. (2011). Belowground Resistance to Western Corn Rootworm in Lepidopteran-Resistant Maize Genotypes. J. Econ. Entomol..

[38] Castano-Duque L., Loades K.W., Tooker J.F., Brown K.M., Paul Williams W., Luthe D.S. (2017). A Maize Inbred Exhibits Resistance Against Western Corn Rootworm, *Diabrotica virgifera virgifera*. J. Chem. Ecol..

[39] Varsani S., Basu S., Williams W.P., Felton G.W., Luthe D.S., Louis J. (2016). Intraplant Communication in Maize Contributes to Defense against Insects. Plant Signal. Behav..

[40] Varsani S., Grover S., Zhou S., Koch K.G., Huang P.-C., Kolomiets M.V., Williams W.P., Heng-Moss T., Sarath G., Luthe D.S. (2019). 12-Oxo-Phytodienoic Acid Acts as a Regulator of Maize Defense against Corn Leaf Aphid. Plant Physiol..

[41] Pingault L., Basu S., Zogli P., Williams W.P., Palmer N., Sarath G., Louis J. (2021). Aboveground Herbivory Influences Belowground Defense Responses in Maize. Front. Ecol. Evol..

[42] Mohan S., Ma P.W.K., Williams W.P., Luthe D.S. (2008). A Naturally Occurring Plant Cysteine Protease Possesses Remarkable Toxicity against Insect Pests and Synergizes *Bacillus thuringiensis* Toxin. PLoS ONE.

[43] Paddock K.J., Robert C.A.M., Erb M., Hibbard B.E. (2021). Western Corn Rootworm, Plant and Microbe Interactions: A Review and Prospects for New Management Tools. Insects.

[44] Castano-Duque L., Luthe D.S. (2018). Protein Networks Reveal Organ-Specific Defense Strategies in Maize in Response to an Aboveground Herbivore. Arthropod-Plant Interact..

[45] Pechan T., Cohen A., Williams W.P., Luthe D.S. (2002). Insect Feeding Mobilizes a Unique Plant Defense Protease That Disrupts the Peritrophic Matrix of Caterpillars. Proc. Natl. Acad. Sci. USA.

[46] Nguyen D., Rieu I., Mariani C., van Dam N.M. (2016). How Plants Handle Multiple Stresses: Hormonal Interactions Underlying Responses to Abiotic Stress and Insect Herbivory. Plant Mol. Biol..

[47] Wang K.L.-C., Li H., Ecker J.R. (2002). Ethylene Biosynthesis and Signaling Networks. Plant Cell.

[48] Li W., Ma M., Feng Y., Li H., Wang Y., Ma Y., Li M., An F., Guo H. (2015). EIN2-Directed Translational Regulation of Ethylene Signaling in Arabidopsis. Cell.

[49] Ogunola O.F., Hawkins L.K., Mylroie E., Kolomiets M.V., Borrego E., Tang J.D., Williams W.P., Warburton M.L. (2017). Characterization of the Maize Lipoxygenase Gene Family in Relation to Aflatoxin Accumulation Resistance. PLoS ONE.

[50] Woldemariam M.G., Ahern K., Jander G., Tzin V. (2018). A Role for 9-Lipoxygenases in Maize Defense against Insect Herbivory. Plant Signal Behav..

[51] Mueller M.J. (1997). Enzymes Involved in Jasmonic Acid Biosynthesis. Physiol. Plant.

[52] Stenzel I., Hause B., Maucher H., Pitzschke A., Miersch O., Ziegler J., Ryan C.A., Wasternack C. (2003). Allene Oxide Cyclase Dependence of the Wound Response and Vascular Bundle-Specific Generation of Jasmonates in Tomato—Amplification in Wound Signalling. Plant J..

[53] Staswick P.E., Tiryaki I. (2004). The Oxylipin Signal Jasmonic Acid Is Activated by an Enzyme That Conjugates It to Isoleucine in Arabidopsis. Plant Cell.

[54] Yan Y., Christensen S., Isakeit T., Engelberth J., Meeley R., Hayward A., Emery R.J.N., Kolomiets M.V. (2012). Disruption of *OPR7* and *OPR8* Reveals the Versatile Functions of Jasmonic Acid in Maize Development and Defense. Plant Cell.

[55] Shivaji R., Camas A., Ankala A., Engelberth J., Tumlinson J.H., Williams W.P., Wilkinson J.R., Luthe D.S. (2010). Plants on Constant Alert: Elevated Levels of Jasmonic Acid and Jasmonate-Induced Transcripts in Caterpillar-Resistant Maize. J. Chem. Ecol..

[56] Dempsey D.A., Vlot A.C., Wildermuth M.C., Klessig D.F. (2011). Salicylic Acid Biosynthesis and Metabolism. Arab. Book.

[57] Lefevere H., Bauters L., Gheysen G. (2020). Salicylic Acid Biosynthesis in Plants. Front. Plant Sci..

[58] Dutartre L., Hilliou F., Feyereisen R. (2012). Phylogenomics of the Benzoxazinoid Biosynthetic Pathway of Poaceae: Gene Duplications and Origin of the Bx Cluster. BMC Evol. Biol..

[59] Niemeyer H.M. (1988). Hydroxamic Acids (4-Hydroxy-1,4-Benzoxazin-3-Ones), Defence Chemicals in the Gramineae. Phytochemistry.

[60] Sicker D., Frey M., Schulz M., Gierl A. (2000). Role of Natural Benzoxazinones in the Survival Strategy of Plants. Int. Rev. Cytol..

[61] Jonczyk R., Schmidt H., Osterrieder A., Fiesselmann A., Schullehner K., Haslbeck M., Sicker D., Hofmann D., Yalpani N., Simmons C. (2008). Elucidation of the Final Reactions of DIMBOA-Glucoside Biosynthesis in Maize: Characterization of *Bx6* and *Bx7*. Plant Physiol..

[62] Woodhouse M.R., Cannon E.K., Portwood J.L., Harper L.C., Gardiner J.M., Schaeffer M.L., Andorf C.M. (2021). A Pan-Genomic Approach to Genome Databases Using Maize as a Model System. BMC Plant Biol..

[63] Erb M., Flors V., Karlen D., de Lange E., Planchamp C., D’Alessandro M., Turlings T.C.J., Ton J. (2009). Signal Signature of Aboveground-Induced Resistance upon Belowground Herbivory in Maize. Plant J..

[64] Baldwin I.T., Schmelz E.A., Ohnmeiss T.E. (1994). Wound-Induced Changes in Root and Shoot Jasmonic Acid Pools Correlate with Induced Nicotine Synthesis In *Nicotiana sylvestris* Spegazzini and Comes. J. Chem. Ecol..

[65] Chapman K.M., Marchi-Werle L., Hunt T.E., Heng-Moss T.M., Louis J. (2018). Abscisic and Jasmonic Acids Contribute to Soybean Tolerance to the Soybean Aphid (*Aphis glycines* Matsumura). Sci. Rep..

[66] Liu L., Sonbol F.-M., Huot B., Gu Y., Withers J., Mwimba M., Yao J., He S.Y., Dong X. (2016). Salicylic Acid Receptors Activate Jasmonic Acid Signalling through a Non-Canonical Pathway to Promote Effector-Triggered Immunity. Nat. Commun..

[67] Ye W., Bustos-Segura C., Degen T., Erb M., Turlings T.C.J. (2022). Belowground and Aboveground Herbivory Differentially Affect the Transcriptome in Roots and Shoots of Maize. Plant Direct.

[68] Robert C.A.M., Veyrat N., Glauser G., Marti G., Doyen G.R., Villard N., Gaillard M.D.P., Köllner T.G., Giron D., Body M. (2012). A Specialist Root Herbivore Exploits Defensive Metabolites to Locate Nutritious Tissues. Ecol. Lett..

[69] Robert C.A., Zhang X., Machado R.A., Schirmer S., Lori M., Mateo P., Erb M., Gershenzon J. (2017). Sequestration and Activation of Plant Toxins Protect the Western Corn Rootworm from Enemies at Multiple Trophic Levels. eLife.

[70] Schnepf H.E., Lee S., Dojillo J., Burmeister P., Fencil K., Morera L., Nygaard L., Narva K.E., Wolt J.D. (2005). Characterization of Cry34/Cry35 Binary Insecticidal Proteins from Diverse *Bacillus thuringiensis* Strain Collections. Appl. Environ. Microbiol..

[71] Ellis R.T., Stockhoff B.A., Stamp L., Schnepf H.E., Schwab G.E., Knuth M., Russell J., Cardineau G.A., Narva K.E. (2002). Novel *Bacillus Thuringiensis* Binary Insecticidal Crystal Proteins Active on Western Corn Rootworm, *Diabrotica virgifera virgifera* LeConte. Appl. Environ. Microbiol..

[72] Wang H., Eyun S.-I., Arora K., Tan S.Y., Gandra P., Moriyama E., Khajuria C., Jurzenski J., Li H., Donahue M. (2017). Patterns of Gene Expression in Western Corn Rootworm (*Diabrotica virgifera virgifera*) Neonates, Challenged with Cry34Ab1, Cry35Ab1 and Cry34/35Ab1, Based on Next-Generation Sequencing. Toxins.

[73] Sparks M.E., Blackburn M.B., Kuhar D., Gundersen-Rindal D.E. (2013). Transcriptome of the *Lymantria dispar* (Gypsy Moth) Larval Midgut in Response to Infection by *Bacillus thuringiensis*. PLoS ONE.

[74] Lei Y., Zhu X., Xie W., Wu Q., Wang S., Guo Z., Xu B., Li X., Zhou X., Zhang Y. (2014). Midgut Transcriptome Response to a Cry Toxin in the Diamondback Moth, *Plutella xylostella* (Lepidoptera: Plutellidae). Gene.

[75] Ritchie J.T., Singh U., Godwin D.C., Bowen W.T., Tsuji G.Y., Hoogenboom G., Thornton P.K. (1998). Cereal Growth, Development and Yield. Understanding Options for Agricultural Production.

[76] Ankala A., Kelley R.Y., Rowe D.E., Williams W.P., Luthe D.S. (2013). Foliar Herbivory Triggers Local and Long Distance Defense Responses in Maize. Plant Sci..

[77] Andrews S. FastQC: A Quality Control Tool for High Throughput Sequence Data. https://www.bioinformatics.babraham.ac.uk/projects/fastqc/.

[78] Bolger A.M., Lohse M., Usadel B. (2014). Trimmomatic: A Flexible Trimmer for Illumina Sequence Data. Bioinformatics.

[79] Kim D., Pertea G., Trapnell C., Pimentel H., Kelley R., Salzberg S.L. (2013). TopHat2: Accurate Alignment of Transcriptomes in the Presence of Insertions, Deletions and Gene Fusions. Genome Biol..

[80] Langfelder P., Horvath S. (2008). WGCNA: An R Package for Weighted Correlation Network Analysis. BMC Bioinform..

[81] Pingault L., Varsani S., Palmer N., Ray S., Williams W.P., Luthe D.S., Ali J.G., Sarath G., Louis J. (2021). Transcriptomic and Volatile Signatures Associated with Maize Defense against Corn Leaf Aphid. BMC Plant Biol..

[82] Lin W.-D., Chen Y.-C., Ho J., Hsiao C. (2006). GOBU: Toward an Integration Interface for Biological Objects. J. Inf. Sci. Eng..

