# Supplementary material for: Co-Transcriptomic Analysis of the Maize–Western Corn Rootworm Interaction
Source: Plants (Basel). 2022 Sep 7;11(18):2335. doi: 10.3390/plants11182335 (PMC9505089; doi:10.3390/plants11182335)
Supplement: Supplementary file 1 [file plants-11-02335-s001.zip › plants-1741276-supplementary/supplementary Figure S1.pdf]

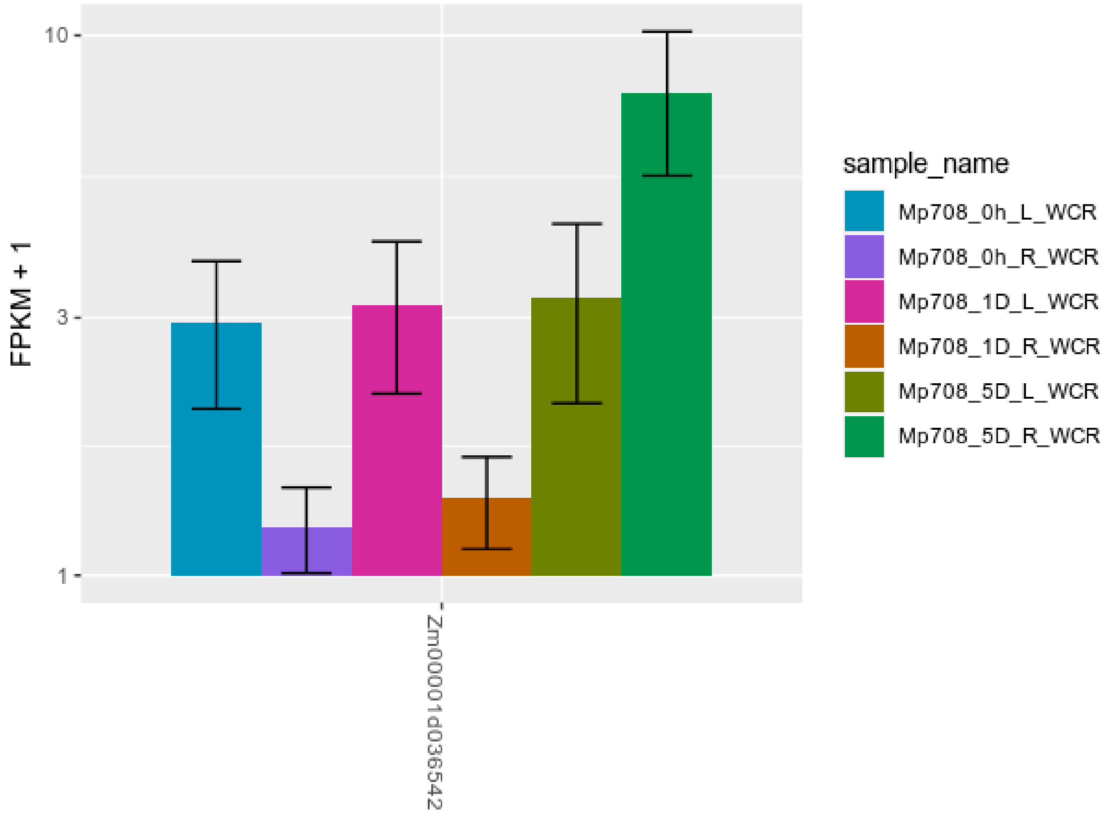


**Supplemental Figure S1**: The expression level of *mir1* (*Zm00001d036542*) across each maize conditions. L: leaves, R: roots, WCR: western corn rootworm.
